# Outstanding Reviewers for *Chemical Science* in 2021

**DOI:** 10.1039/d2sc90113g

**Published:** 2022-07-08

## Abstract

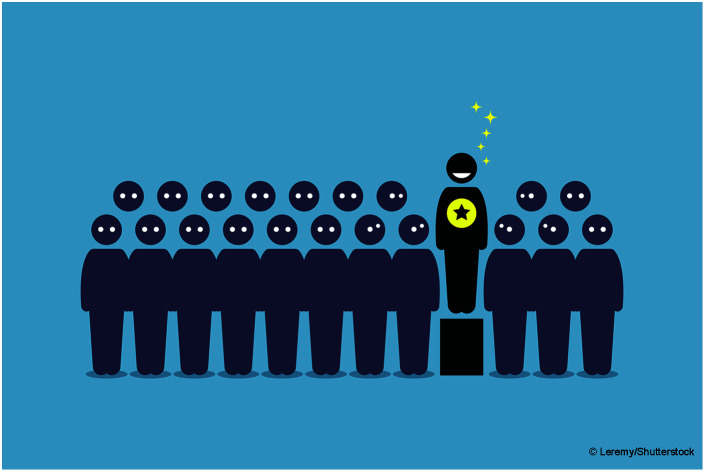

We would like to take this opportunity to thank all of *Chemical Science*’s reviewers, and in particular highlight the Outstanding Reviewers for the journal in 2021, as selected by the editorial team for their significant contribution to *Chemical Science*. We announce our Outstanding Reviewers annually and each receives a certificate to give recognition for their contribution.

Our list includes reviewers who provided a much higher than average number of excellent quality reports. However, we recognize that this is not the only important measure, so we are also highlighting reviewers who provided extraordinarily detailed reports, reviewers who were particularly noted for constructively helping authors to improve their manuscripts, and reviewers who provided noteworthy and thoughtful adjudicative reports as well. By widening our selection criteria, our aim is to provide a more diverse and representative sample of our reviewer community.

Celebrating the diversity and recognizing the efforts of our reviewer community is incredibly important. That is why, in addition to our yearly Outstanding Reviewer editorial, we also highlight a selection of reviewers each month in our new Reviewer Spotlight blog. Find out more at https://blogs.rsc.org/sc/category/reviewer-spotlight/ and keep an eye out on social media for our next spotlighted reviewer!

Our Outstanding Reviewers for *Chemical Science* in 2021 are:

 

Dr Diego Andrada

Universität des Saarlandes

ORCID: 0000-0003-2515-7859

 

Dr Goran Angelovski

Chinese Academy of Science

ORCID: 0000-0002-8883-2631

 

Dr Stéphanie Bastin

Université de Toulouse

ORCID: 0000-0002-7512-7423

 

Dr Ashraf Brik

Technion-Israel Institute of Technology

ORCID: 0000-0001-8745-2250

 

Dr Yun Chen

Nanjing Medical University

ORCID: 0000-0002-0952-8851

 

Professor Marcetta Darensbourg

Texas A&M University

ORCID: 0000-0002-0070-2075

 

Professor Nan-Nan Deng

Shanghai Jiao Tong University

ORCID: 0000-0001-7183-7973

 

Dr Elisa Fadda

National University of Ireland Maynooth

ORCID: 0000-0002-2898-7770

 

Dr Jeremiah Gassensmith

University of Texas at Dallas

ORCID: 0000-0001-6400-8106

 

Professor Jonathan Goodman

University of Cambridge

ORCID: 0000-0002-8693-9136

 

Professor Shigeyoshi Inoue

Technische Universitat Munchen

ORCID: 0000-0001-6685-6352

 

Dr Kjell Jorner

University of Toronto

ORCID: 0000-0002-4191-6790

 

Dr Marina Kuimova

Imperial College London

ORCID: 0000-0003-2383-6014

 

Professor Daniele Leonori

RWTH Aachen University

ORCID: 0000-0002-7692-4504

 

Professor Christine Luscombe

Okinawa Institute of Science and Technology

ORCID: 0000-0001-7456-1343

 

Professor Satoshi Maeda

Hokkaido University

ORCID: 0000-0001-8822-1147

 

Dr Tarun Panda

Indian Institute of Technology Hyderabad

ORCID: 0000-0003-0975-0118

 

Dr Sofia Pauleta

Universidade Nova de Lisboa

ORCID: 0000-0002-2149-9416

 

Dr Samuel Sanders

Rowland Institute at Harvard

 

Dr Sayaka Uchida

University of Tokyo

 

Dr Matthias Wagner

Goethe University Frankfurt

ORCID: 0000-0001-5806-8276

 

Professor Jianfang Wang

Chinese University of Hong Kong

ORCID: 0000-0002-2467-8751

 

Dr Jiang Weng

Sun Yat-sen University

ORCID: 0000-0002-4117-4686

 

Professor Peng Yang

Shaanxi Normal University

ORCID: 0000-0002-0463-1024

 

Dr Sangwoon Yoon

Chung-Ang University

ORCID: 0000-0001-5705-4569

 

We would also like to thank the *Chemical Science* Editorial Board and Advisory Board and the chemical science community for their continued support of the journal, as authors, reviewers and readers.

 

May Copsey, Executive Editor

## Supplementary Material

